# Differences in leadership ambition of women and men in their early academic career in cardiovascular research in the Netherlands

**DOI:** 10.1007/s12471-026-02025-x

**Published:** 2026-02-26

**Authors:** Birgit Goversen, Elise L. Kessler, Linda Modderkolk, Sabine Oertelt-Prigione, Hester M. den Ruijter

**Affiliations:** 1 Department of Physiology, Amsterdam Cardiovascular Sciences, Amsterdam University Medical Centres, Amsterdam, The Netherlands; 2 3Rs Centre Utrecht, Animal Welfare Body Utrecht, Utrecht University, Utrecht, The Netherlands; 3 Laboratory of Experimental Cardiology, Department of Cardiology, University Medical Centre Utrecht, Utrecht University, Utrecht, The Netherlands; 4 Anaesthesiology, Pain and Palliative Medicine, Radboud University Medical Centre, Nijmegen, The Netherlands; 5 Department of Primary and Community Care, Radboud University Medical Centre, Nijmegen, The Netherlands

**Keywords:** Cardiology, Research, Leadership, Motivation

## Abstract

In the field of cardiology and cardiovascular research, the underrepresentation of female leaders persists despite efforts to promote equality. As this phenomenon could have various reasons, we investigated the influence of intrinsic and extrinsic motivation on the representation of women in leadership roles. In this pilot study, we explored the motivation of Dutch cardiovascular PhD candidates for pursuing leadership positions through a survey. Among 143 respondents (97 female, 46 male), family planning did not seem to impact leadership ambitions as much as the desire to work part-time in the future. Of the participating women, 12% stated that a female quota would encourage them to pursue a leadership position. Interestingly, communal traits, which are typically associated with femininity, were perceived as hampering towards leadership by women but not by men. Our results show that gender stereotypes continue to influence cardiology careers, and that initiatives aimed at overall cultural change may be more supportive for future female leaders than single policy measures.

## What’s new?

- In this pilot study we explored the motivation for leadership of PhD candidates in cardiovascular research in the Netherlands
- Our results reveal that not family planning, but the desire to work part-time is negatively associated with the leadership aspirations of women
- Communal traits, generally linked to stereotypical female traits, such as ‘being humble’ are considered as hampering for a leader by women, but not by men

## Introduction

Despite years of advancement in gender equality initiatives in academia in general and specifically in medicine, women remain underrepresented in leadership positions, especially in the field of cardiology and cardiovascular research (in 2023, 22.9% of Dutch cardiologists were female) [1]. This gender bias could arise from work-family balance challenges, the persistence of the glass ceiling, or the effects of specific leadership traits [2, 3]. In academia, communal character traits—stereotypically linked to women—are seen as hampering success as compared to agentic traits, which are stereotypically linked to men [3]. Work-life balance does not appear as the primary reason, given that women in the Netherlands work part-time even before family planning, representing the largest share of women working part-time in the European Union (in 2022, 63% women and 24% men) [4]. Therefore, understanding the interplay of these multiple factors is essential for explaining the persistent underrepresentation of women in leadership roles. To identify gender differences in leadership ambition, we inquired into which personal/professional factors and leadership perceptions influence young cardiovascular (bio)medical professionals’ motivation to pursue leadership positions.

## Methods

In the years 2018 and 2019, we surveyed Dutch cardiovascular PhD candidates during national research courses and through their research institutes with an online questionnaire. We asked PhD candidates questions about their demographics (gender, age, marital status etc.), their work, personal and professional future planning, and leadership aspirations in 36 multiple-choice and 12 open questions. The respondents (97 female and 46 male) were asked to rate work-related initiatives that promote pursuing a leadership position (on a scale from not motivating to very motivating), and specific character traits for a leader (on a scale from hampering to beneficial), analysed by chi-square (using SPSS software). PhD candidates were also asked to identify their own traits they considered hampering and beneficial for leadership in open questions. We scored the responses as communal (100%) or agentic (0%), averaged per person for continuous data transformation, and analysed by two-way Analysis of Variance (using Prism software). Data normality was assessed using Shapiro-Wilk, and a *p*-value ≤ 0.05 was deemed statistically significant.

## Results

Our survey was filled out by 143 respondents, with a mean age of 25–29 years old (107 of 143 individuals), and 68% were women. Out of 143 respondents, 57% of the women and 68% of the men aim for a leadership position in the future, defined as e.g., professor, (medical) group leader, dean, (medical) director, manager, corporate executive officer, or corporate partner (Tab. 1). We did not observe gender differences in plans to work part-time, with 72% of the women indicating the desire to work part-time in the future versus 63% of the men.

**Table 1:** Baseline characteristics, personal and professional future planning, and leadership aspirations of volunteer PhD candidates who responded in this study

	Women	Men
*Baseline characteristics*		
*n* (%)	97 (68)	46 (32)
Age, mean (range)	25–29	25–29
Civil status		
Married/in a relationship *n* (%)	68 (70)	35 (76)
Single *n* (%)	29 (30)	11 (24)
*Plans for future*		
Plan to work part-time *n* (%)		
Yes	70 (72)	29 (63)
Maybe	12 (12)	6 (13)
No	16 (16)	11 (24)
Plan to have children *n* (%)		
Yes	52 (54)	28 (61)
Maybe	23 (24)	6 (13)
No	16 (16)	7 (15)
I already have children, yes	6 (6)	5 (11)
Aim for leadership position *n* (%)		
Yes	56 (57)	31 (68)
Maybe	24 (25)	7 (15)
No	18 (18)	8 (17)

Results are presented as frequencies (percentages %) for women and men

None of the characteristics were statistically significant between women and men

The ambition to have children was indicated by 54% of the women and 61% of the men in our survey, which did not influence leadership aspiration in either women or men. However, the desire to work part-time correlated negatively with the leadership aspiration of women (*p* = 0.04); this correlation did not apply to men.

We did not observe any gender differences in the perception of potentially motivating structural factors. A female quota would motivate 12% of women respondents to pursue a leadership position.

**Fig. 1:**
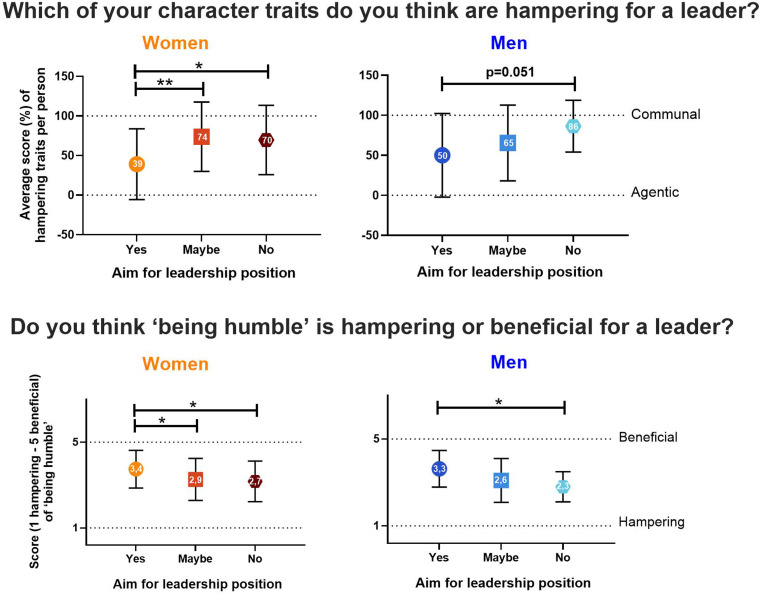
Hampering character traits in leadership. Plot combining “Are you aiming for a leadership position” (yes, maybe, no), with other questions. *Top row:* “Which of your character traits do you think are hampering for a leader?” (communal (100%) and agentic (0%)). *Bottom row:* “Do you think ‘being humble’ is hampering or beneficial for a leader?” (1 = hampering, 5 = beneficial). *Left graph:* women (*n* = 97); *right graph:* men (*n* = 46). Results were transformed into continuous data, presented as mean ± standard deviation, and compared between groups with a Two-way Analysis of Variance with *p < 0.05, **p < 0.005

When asked about their character traits (Fig. 1), women with limited leadership aspirations (no/maybe) perceived their communal traits as more hampering for leadership than women aiming for a leadership position (*p* = 0.034 and *p* = 0.007, respectively). In line with this, women with limited leadership aspirations (no/maybe) think ‘being humble’ is more hampering for a leader than women who aim for a leadership position (‘no’ *p* = 0.015 and ‘maybe’ *p* = 0.046). Male respondents, regardless of their aim for leadership, did not consider their communal traits hampering for leadership, but showed a trend comparable to women (n. s. with *p* = 0.051 for ‘no’). Men only perceived ‘being humble’ as hampering when they had no leadership aspirations (*p* = 0.04).

## Discussion

Our survey reveals that family planning does not seem to impact women’s leadership aspirations in the context of cardiology and cardiovascular research. Interestingly, in recent years, the potential impact of childcare support, such as free day-care, on parents’ health and work ambitions has been extensively researched. It has been shown on various occasions that this is especially important to low-income families and has great effects on the parents’ (mental) health, free time, and therefore professional ambitions [5, 6]. Of the female respondents, 12% would be encouraged by a female quota to pursue a leadership position. This is in line with a social experiment performed by Maggian and colleagues, where a female quota was only effective if women were already very far in the competition [7], highlighting the importance of gender salience (the self-awareness of one’s gender) in decision making. Part-time work appears hardly reconcilable with women’s leadership aspirations. This is very interesting given that the Netherlands holds the largest share of women working part-time in the European Union [4]. Interestingly, a generational change can be seen in the interest in alternative and individualized work options (e.g., part-time and remote) in many fields of work in Generation Z compared to former generations [8]. This might also apply to this study population. Women and men with limited leadership aspirations perceive their communal traits, e.g., being humble, as hampering, albeit women more prominently than men. These findings are in line with the literature, where leadership traits are perceived differently according to gender identity, e.g., a dominant attitude is acceptable for men but negatively connoted for women [9]. Differences among women can be explained by self-categorization theory, which suggests that self-stereotyping decreases when gender salience is low [10]. Therefore, if women realize that gender differences affect their career outcomes, this might increase their self-stereotyping. Although communal traits are considered as beneficial for a leader [11], the idea that men, who more frequently display agentic traits, are more suitable for leadership positions still prevails in society at large [12]. Fortunately, for instance, in the United States, women have been less prone to view management and leadership as the sole domain of men over the last decade [13], and women with a female manager or a high percentage of female leaders seem to value feminine managerial characteristics and female managers over male ones [14]. This underlines the importance of a mixed and diverse work floor.

Overall, the respondents’ replies confirm stereotypical professional attributes in the field of cardiology and cardiovascular research [15]. Gendered social role expectations like professional attributes should be contextualized, as cultural and professional environments are important moderators driving perception and behaviour. Our study highlights once more the importance of one’s awareness of these complex social structures as well as one’s personal influence and (self)perception. We therefore recommend that the education and training of future cardiovascular (bio)medical professionals and researchers emphasize the impact of these issues, promote awareness, and prioritize positive culture change.

## Data Availability

The data that supports the findings of this study are available from the corresponding author (BG), upon reasonable request.
